# Emergency management of doctor–patient group incidents in a regional general hospital based on the 4R crisis management theory

**DOI:** 10.3389/fmed.2026.1887026

**Published:** 2026-10-28

**Authors:** Jingjing Song, Xiantao Huang, Jingfan Zhao, Xiangxin Niu, Xuanbo Jin, Yujun Ren

**Affiliations:** 1 Legal Department, The First Affiliated Hospital of Hebei North University, Zhangjiakou, Hebei, China; 2 Quality Control Department, The First Affiliated Hospital of Hebei North University, Zhangjiakou, Hebei, China; 3 Technology Department, The First Affiliated Hospital of Hebei North University, Zhangjiakou, Hebei, China; 4 Resident Standardised Training Office, The First Affiliated Hospital of Hebei North University, Zhangjiakou, Hebei, China

**Keywords:** 4R crisis management, doctor–patient group incidents, emergency management, hospital risk communication, retrospective case series

## Abstract

Growing clinical complexity and healthcare demand can intensify medical disputes, and patient deaths or severe complications may be followed by collective disruptive events. This single-center retrospective descriptive case series used the 4R crisis management framework (Reduction, Readiness, Response, and Recovery) to organize the review of 10 doctor–patient group incidents documented in a regional general hospital from 2013 to 2023. We summarized incident characteristics, departments, collective behaviors, handling pathways, record-coded contributing factors, and documented hospital activities, and mapped each incident to the four 4R stages using predefined coding rules. All included incidents followed a patient death. Four incidents occurred in pediatrics or neonatology, and two occurred in cardiothoracic surgery. Frequently documented collective behaviors included ward or body retention (9/10), banners or memorial displays (7/10), obstruction of clinicians or offices (5/10), and online dissemination (2/10). The 4R mapping provided a stage-based description of recorded risk communication, preparedness, on-site response, and post-incident follow-up activities. It also supported the development of a proposed organizational framework for future process review. Because the study had no comparison group or standardized response and recovery outcomes, the findings do not establish the effectiveness or causal effects of any management measure.

## 1 Introduction

With increasing demand for healthcare services and the growing complexity of medical technology in China, medical disputes have become important clinical, legal, and policy concerns. They may affect patient safety, staff safety, hospital operations, and institutional reputation (1, 2). Disputes can arise from disagreements about clinical decisions, treatment outcomes, communication, or professional responsibility (3, 4). International concern about attacks on Chinese physicians has also been reported (5).

General hospitals may face collective incidents after patient deaths, severe complications, unsatisfactory outcomes, or allegations of medical error (6). In this study, a doctor–patient group incident was operationally defined as an event arising in a medical-dispute context that involved collective behavior by family members or other participants and either disrupted normal hospital operations or produced externally visible escalation, such as banners, obstruction, police involvement, or online dissemination. The concept overlaps with, but is not identical to, workplace violence, collective protest, and public-opinion events. Workplace violence centers on threats or acts against workers; collective protest may occur outside a medical dispute; and a public-opinion event may occur entirely online. Inclusion in this study required both a medical-dispute context and collective disruption or externally visible escalation.

Traditional approaches that focus mainly on immediate response may not capture activities across the full incident lifecycle. The 4R crisis management framework—Reduction, Readiness, Response, and Recovery—organizes risk mitigation, preparedness, acute response, and post-incident review within one structure (7, 8). Emergency-management literature also emphasizes coordinated multisectoral response, clear command arrangements, adequate preparation, and timely psychological support (9).

Using a regional general hospital as a case setting, this study aimed to describe the characteristics of 10 included doctor–patient group incidents, map documented management activities to the four 4R stages, and develop a proposed stage-based framework for institutional process review. The 4R framework was used as an analytical and organizational structure, not as an instrument for evaluating intervention effectiveness.

## 2 Materials and methods

### 2.1 Study subjects

This single-center retrospective descriptive case series examined doctor–patient group incidents documented in a regional general hospital between 2013 and 2023. For the purpose of this study, a doctor–patient group incident was defined as an event arising in a medical-dispute context that involved collective behavior disrupting hospital operations or producing externally visible escalation. Individual complaints without collective disruption, violence unrelated to a medical dispute, protests unrelated to medical care, and online discussion without a linked collective incident were not considered doctor–patient group incidents under this operational definition. The complete case set was identified through a retrospective review of the medical-dispute case records maintained by the hospital's Legal Department for 2013–2023. The records were retrieved by the first author, and the resulting case list was independently verified by a second researcher against the Legal Department's archived medical-dispute records for the same period. This review identified 10 doctor–patient group incidents meeting the operational definition, all of which were included in the analysis. No sampling or further exclusion of eligible incidents was performed. The study protocol was approved by the hospital ethics committee (Ethical No. L2026001). The records used for analysis were anonymized to protect patient and family privacy, and all cases are reported using numerical identifiers (Case 1–Case 10) rather than patient names or surnames. The remaining combinations of potentially identifying information, including incident date, department, clinical circumstances, processing duration, and compensation amount, were reviewed for re-identification risk before manuscript preparation. Only information considered necessary for the descriptive analysis and consistent with the ethics approval and applicable institutional privacy requirements was retained.

### 2.2 Study design

A retrospective case-study design combined descriptive quantitative summaries with qualitative content analysis. The incident was the unit of analysis, and the 4R framework was specified before mapping. The analytical sequence comprised data collection and chronological standardization, descriptive coding, independent qualitative coding, 4R-stage mapping, cross-case synthesis, and development of a proposed management framework. The study did not compare interventions, test causal relationships, or evaluate the effectiveness of response or recovery measures.

### 2.3 Data sources and coding framework

Incident data for all 10 cases were obtained from the corresponding case files maintained by the hospital's Legal Department, including hospital management records, administrative reports, and structured event documentation. Available documents relating to each incident were treated as source materials for that incident and arranged chronologically. The coding domains were: (1) documented precipitating event; (2) department; (3) collective family behaviors; (4) handling pathway, processing time, and compensation as recorded; (5) record-coded contributing factors; (6) documented hospital, mediation, police, psychological-support, and public-opinion-management activities; and (7) the timing of activities in relation to the incident.

A documented precipitating event was the immediate clinical event explicitly stated in the source record, such as death or a severe postoperative complication. A record-coded contributing factor was a concern explicitly documented in an administrative review note or structured event record. These labels were not treated as adjudicated causes, confirmed medical errors, malpractice determinations, or formal root causes. Allegations, compensation payments, mediated settlements, and court involvement were not interpreted as proof of medical fault. Literature-based explanations were excluded from case coding and were used only for contextual interpretation in the Discussion.

The 4R categories were defined before coding. Reduction included documented activities before escalation that concerned risk identification, risk assessment, informed communication, or expectation management. Readiness included emergency plans, teams, training, resource preparation, and early-warning arrangements. Response included actions after the onset of collective disruption, including on-site coordination, security or police involvement, mediation, information communication, and public-opinion monitoring. Recovery included post-acute counseling, communication follow-up, process review, and documented procedural or institutional adjustment.

A management gap was coded only when a source record explicitly described a process as absent, delayed, insufficient, or subsequently corrected. A gap was not inferred from an adverse outcome, compensation amount, processing duration, family behavior, mediation, or police involvement alone.

Two researchers independently reviewed the chronological records, coded the predefined domains, and mapped each incident to the 4R stages. They then checked the coded summary against the source records and resolved disagreements through consensus discussion. A third researcher adjudicated unresolved differences. No inter-rater agreement statistic was calculated because the sample was small and the analysis was descriptive.

### 2.4 Data analysis

#### 2.4.1 Quantitative analysis

Counts, percentages, ranges, and descriptive summaries were calculated for incident characteristics, departmental distribution, handling pathways, collective behaviors, and record-coded contributing factors. Because the series included only 10 incidents, no inferential, association, correlation, or trend analysis was performed. Figures were produced in R version 4.3.2 using ggplot2.

#### 2.4.2 Qualitative analysis

Content analysis followed a predefined sequence of chronological review, independent coding, 4R mapping, cross-case summary, source-record checking, consensus discussion, and third-researcher adjudication when required. The mapping characterized the recorded activities and their timing. It did not attribute incident resolution or any subsequent outcome to a specific activity.

#### 2.4.3 Model construction

The proposed 4R-based framework was developed through narrative synthesis of the descriptive and qualitative findings. It organizes possible tasks, resources, and responsible units across the four stages. The framework was not prospectively implemented, validated, or tested for effectiveness in this study.

## 3 Results

### 3.1 General characteristics of doctor–patient group incidents

The 10 included incidents varied in processing time and recorded compensation amount (Figure 1). The documented precipitating events and collective manifestations of the 10 included incidents are summarized in Table 1. Processing time ranged from 7 days to 647 days, and compensation ranged from RMB 5,000 to RMB 1,050,000.

**Figure 1:**
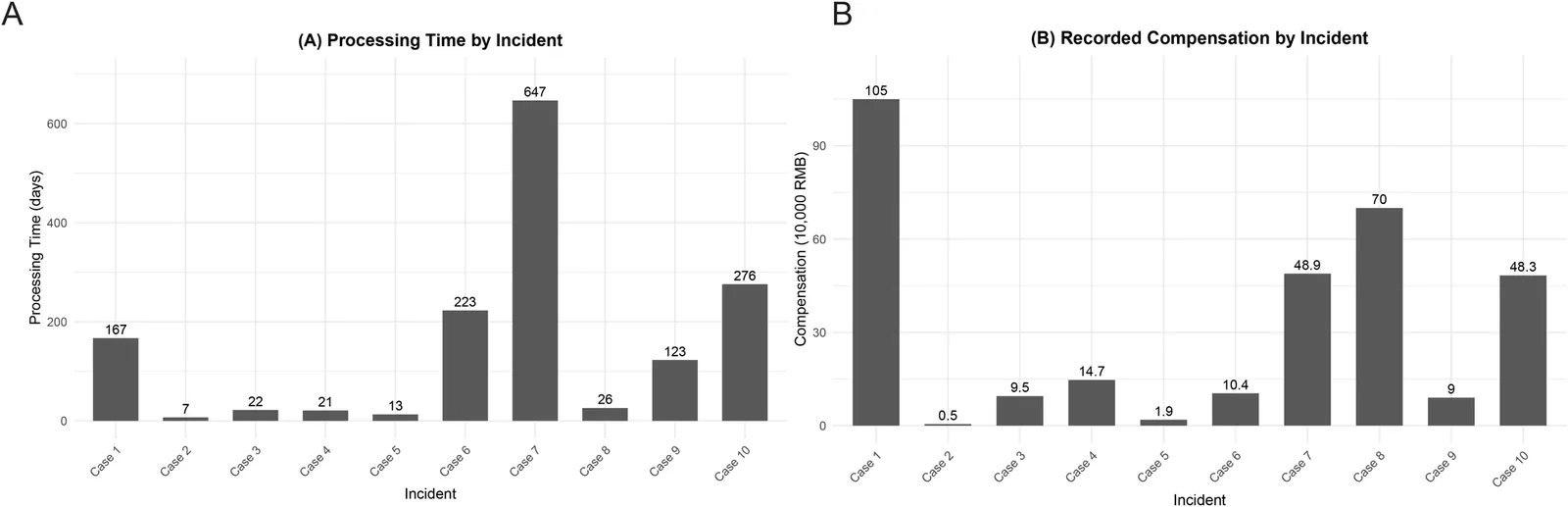
Recorded processing time and compensation by incident. **(A)** Processing time by incident; **(B)** recorded compensation by incident. The two variables are presented separately for descriptive purposes only; no correlation or other association between processing time and compensation was tested.

**Table 1:** Documented precipitating events and collective manifestations in the 10 included incidents.

No.	Case	Date	Department	Documented precipitating event	Collective manifestation
1	Case 1	Feb 2013	Trauma Surgery	Patient experienced sudden death 20 days after brain hemorrhage surgery	The family retained the body in the ward, displayed white banners at the hospital entrance, and burned paper inside the hospital.
2	Case 2	Dec 2014	Pediatrics	Child hospitalized for fever and respiratory distress, died suddenly during x-ray examination outside the ward with parental accompaniment	The family disrupted the outpatient process and displayed banners outside the outpatient area; police involvement was recorded.
3	Case 3	Feb 2015	Obstetrics	Infant died 4 h after cesarean section	The family refused transfer of the infant's body to the mortuary and disrupted normal ward operations; police involvement was recorded.
4	Case 4	Feb 2016	Orthopedics	Patient died due to postoperative bleeding; RH(-) blood type lacked compatible transfusion	The family repeatedly obstructed the director's office; police and mediation committee involvement was recorded.
5	Case 5	Nov 2016	Cardiothoracic Surgery ICU	Patient died after mitral valve replacement + radiofrequency ablation due to cardiac dysfunction	The family refused transfer of the body to the mortuary and retained it in the ICU; police involvement was recorded.
6	Case 6	Feb 2017	Cardiothoracic Surgery	Patient died from massive bleeding during prolonged aortic valve replacement with extracorporeal circulation	The family caused disruption in the outpatient area; police involvement was recorded.
7	Case 7	Jul 2018	Neonatology	Infant died 5 days after birth due to jaundice and umbilical infection	The family displayed white banners and a memorial photograph; police and mediation committee involvement was recorded.
8	Case 8	Jun 2020	Dentistry	Child developed sudden respiratory distress after sublingual gland cyst excision	The family remained in the ICU, wore mourning clothes, displayed a memorial photograph, and disseminated videos online; police involvement was recorded.
9	Case 9	Feb 2021	Pediatrics	Child hospitalized for respiratory distress, condition deteriorated during hospitalization	The family repeatedly obstructed the director's office and interfered with clinical work; police and mediation committee involvement was recorded.
10	Case 10	Jan 2022	Pediatrics	Child visited outpatient for abdominal discomfort and vomiting; died suddenly during observation	The family repeatedly obstructed clinicians and disrupted clinical operations; police involvement was recorded.

The precipitating event is the immediate event documented in the source record and does not represent an adjudicated cause or a confirmed medical error.

The 2018 incident had the longest processing time (647 days) and a recorded compensation amount of RMB 489,000, whereas the 2013 incident had the highest recorded compensation amount (RMB 1,050,000) and a processing time of 167 days. These values provide a descriptive comparison only; no correlation between processing time and compensation was tested.

The recorded handling pathway was mediation committee coordination in 4 incidents (40%), court handling in 3 incidents (30%), and self-negotiation in 3 incidents (30%). These frequencies describe the included cases and do not compare the outcomes of the three pathways.

Ward or body retention was documented in 9 incidents (90%), banners or memorial displays in 7 incidents (70%), obstruction of a director, physician, or office in 5 incidents (50%), and online dissemination in 2 incidents (20%). More than one behavior could occur in the same incident.

### 3.2 Record-coded contributing factors and departmental distribution

Pediatrics accounted for 3 incidents (30%), and Neonatology accounted for 1 incident (10%); when grouped, pediatric and neonatal services accounted for 4 incidents (40%). Cardiothoracic Surgery, including the cardiothoracic surgery ICU, accounted for 2 incidents (20%). Trauma Surgery, Obstetrics, Orthopedics, and Dentistry each accounted for 1 incident (10%). These are counts within the included series; no department-level risk comparison was performed.

The most frequently recorded contributing-factor label was “Inadequate or Overly Optimistic Preoperative Communication” (7 incidents). “Delay in Treatment” and “Inadequate Monitoring of the Patient's Condition” were each recorded in 6 incidents. “Delayed Communication of Changes in the Patient's Condition”, “Insufficient Clinical Attention” and “Inadequate Availability of Blood Products or Equipment” were each recorded in 5 incidents. Other labels occurred in 1–4 incidents (Figure 2). Because more than one factor could be coded for an incident, the total number of factor codes exceeded 10.

**Figure 2:**
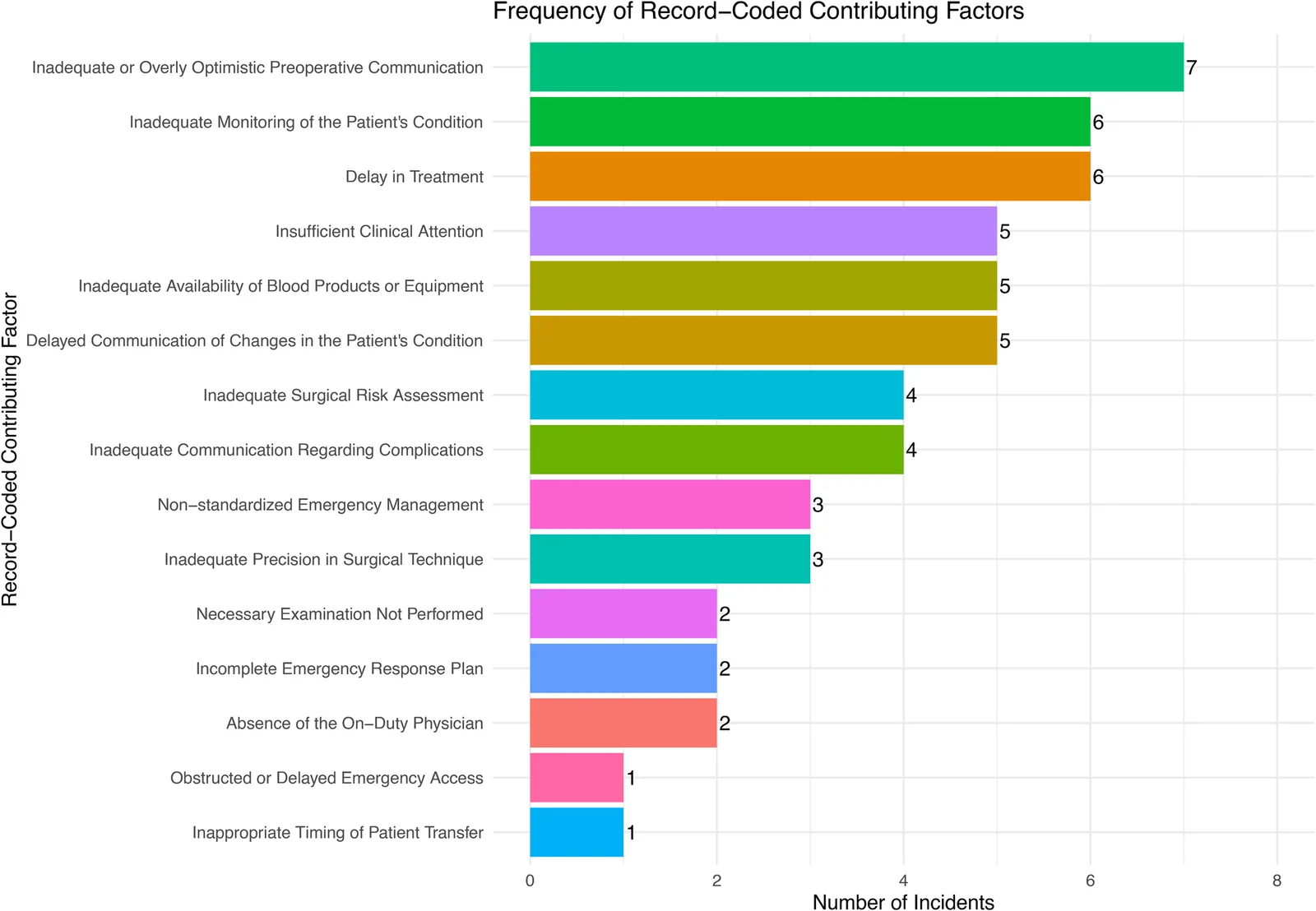
Frequency of record-coded contributing factors in the 10 included incidents. Multiple factors could be coded for one incident. The English labels are translations of categories documented in the original administrative records and are presented as recorded concerns only; they should not be interpreted as adjudicated causes, confirmed medical errors, malpractice determinations, or formal root causes.

These labels reproduce categories found in the administrative records. They are presented as documented concerns and should not be interpreted as adjudicated causes, confirmed medical errors, malpractice determinations, or formal root causes.

### 3.3 Incident response and 4R-stage mapping

The 10 incidents were mapped to the four 4R stages using the predefined coding rules (Table 2). Reduction-stage documentation included risk notification, condition explanation, communication, or assessment. Readiness-stage documentation included emergency teams, emergency plans, training, and resource preparation. Response-stage documentation included collective behaviors at the scene and recorded hospital, mediation, security, or police actions. Recovery-stage documentation included counseling, communication follow-up, public-opinion management, process review, or institutional adjustment. The mapping describes recorded activities and timing only. No standardized response or recovery outcomes were available, and the mapping does not show that any activity caused resolution, restored relationships, prevented recurrence, or improved patient or staff outcomes.

**Table 2:** 4R-stage mapping of documented activities in the 10 included incidents.

Case No.	Case	Dept.	Reduction	Readiness	Response	Recovery
1	Case 1 (2013)	Trauma	Risk notification	Emergency team	Body retained; police involved	Communication follow-up; process review
2	Case 2 (2014)	Pediatrics	Condition notification	Training; resources	Ward disruption; banners; police involved	Counseling; system review
3	Case 3 (2015)	Obstetrics	Preoperative communication	Emergency plan	Body-transfer refusal; police involved	Family communication review
4	Case 4 (2016)	Orthopedics	Risk assessment	Training; emergency team	Office obstruction; police and mediation involved	Process review; psychological support
5	Case 5 (2016)	Cardiothoracic ICU	Surgical risk notification	Emergency team	ICU body retention; police involved	Psychological support; system adjustment
6	Case 6 (2017)	Cardiothoracic	Preoperative communication	Training; resources	Outpatient disruption; police involved	Process review
7	Case 7 (2018)	Neonatology	Neonatal condition notification	Training; emergency plan	Banners; memorial display; police and mediation involved	Communication process review
8	Case 8 (2020)	Dentistry	Surgical risk notification	Emergency preparedness	ICU retention; online dissemination; police involved	Psychological support; public-opinion management
9	Case 9 (2021)	Pediatrics	Condition notification	Emergency team	Office obstruction; police and mediation involved	Process review; communication follow-up
10	Case 10 (2022)	Pediatrics	Preoperative communication	Training; resources	Clinician obstruction; police involved	Psychological support; process review

The mapping describes documented activities and timing only. A management gap was coded only when a source record explicitly described an absent, delayed, insufficient, or subsequently corrected process. No gap or intervention effect was inferred from an adverse outcome, compensation, processing duration, family behavior, mediation, or police involvement alone.

### 3.4 Proposed framework

The cross-case synthesis was used to construct a proposed stage-based organizational framework. The Reduction stage includes risk identification, risk assessment, clear communication of uncertainty, and expectation management. The Readiness stage includes emergency plans, role assignment, training, contact lists, and resource preparation. The Response stage includes on-site command, cross-departmental coordination, security and police liaison when required, mediation, factual information communication, and public-opinion monitoring. The Recovery stage includes psychological support, communication follow-up, case review, and documented procedural or institutional adjustment. This framework is an organizational proposal derived from the case review; its implementation and effectiveness were not evaluated.

## 4 Discussion

This study provides a descriptive account of 10 doctor–patient group incidents documented in one regional general hospital. All included incidents followed a patient death. Four occurred in pediatrics or neonatology, and two occurred in cardiothoracic surgery. These counts describe the included series and do not establish that any department had a higher underlying risk. Previous studies have reported that disputes and malpractice claims may be concentrated in specialties involving severe illness, uncertain prognosis, or complex procedures (10–13), and pediatric adverse events may place substantial psychological stress on families (14). Those studies provide context but do not convert the departmental counts in this case series into risk estimates.

The distinction between case-derived observations and broader interpretation is important. The included records documented precipitating events, family behaviors, management activities, handling pathways, and administrative concern labels. They did not provide a uniform formal root-cause analysis or a medical-malpractice determination for every incident. Accordingly, a recorded allegation, compensation payment, mediated settlement, or court process was not treated as proof of error or malpractice. Explanations involving communication, expectation management, organizational coordination, or resource constraints are discussed as possible contextual mechanisms from the literature, not as confirmed causes of individual incidents.

Ward or body retention, banners or memorial displays, obstruction of clinicians or offices, and online dissemination were the most frequently documented collective behaviors in this series. These observations are descriptive and cannot be generalized beyond the included cases. Research on violence and other serious responses to medical disputes in China has described overlapping forms of disruption (15, 16). Studies of social-media communication further suggest that online dissemination can amplify attention and negative emotion around medical disputes (17–20). This literature supports including public-opinion monitoring and factual information communication in preparedness plans, but the two online cases in this study are insufficient to estimate the magnitude of that effect.

The main contribution of the 4R framework in this study is analytical organization rather than evidence of effectiveness. Mapping records to Reduction, Readiness, Response, and Recovery made it possible to separate activities that occurred before escalation, during preparation, during acute disruption, and after the acute phase. This stage-based review is consistent with crisis-lifecycle approaches that emphasize early identification and preparation (21). However, a management gap was recorded only when the source documentation explicitly described an absent, delayed, insufficient, or subsequently corrected process; gaps were not inferred from the adverse outcome or family behavior alone.

During the Response stage, the records most often described mediation, security or police involvement, and cross-departmental coordination. During the Recovery stage, records referred to counseling, communication follow-up, public-opinion management, process review, or institutional adjustment. Crisis-communication theory and organizational-learning literature emphasize coordinated action and post-crisis review (22, 23), but the present study had no standardized measures of time to control, perceived fairness, psychological recovery, relationship repair, recurrence, or patient and staff outcomes. It therefore cannot determine whether the documented activities were effective or whether they caused the observed end of an incident.

The proposed framework suggests practical areas for future institutional work: standardized communication of treatment uncertainty, explicit escalation criteria, clearly assigned emergency roles, rapid access to mediation and psychological support, procedures for factual public communication, and structured post-incident review. Evidence from medical-dispute appraisal studies also supports careful preoperative assessment and informed consent (24). These recommendations should be prospectively implemented and evaluated with predefined process and outcome measures before conclusions are drawn about benefit.

### 4.1 Limitations

This study has several limitations. First, it was a single-center retrospective descriptive case series with only 10 incidents, which limits transferability and precludes inferential or department-level risk analysis. Second, the completeness and detail of historical records varied, and some activities may not have been documented. Third, coding involved interpretation despite predefined definitions, and no inter-rater agreement statistic was calculated. Fourth, the records did not contain standardized response or recovery outcomes, a comparison group, or prospective follow-up. The study therefore cannot establish causality, compare management approaches, determine intervention effectiveness, or assess whether any measure restored relationships, prevented recurrence, or improved patient or staff outcomes. Multicenter prospective studies with transparent coding rules and predefined outcomes are needed to examine the applicability and utility of the 4R framework.

## 5 Conclusion

This single-center retrospective descriptive case series used the 4R framework to organize documented management activities for 10 doctor–patient group incidents. All included incidents followed a patient death; 4 occurred in pediatrics or neonatology, and 2 occurred in cardiothoracic surgery. Ward or body retention, banners or memorial displays, obstruction of clinicians or offices, and online dissemination were the principal collective behaviors recorded. The 4R mapping separated documented activities into risk reduction, readiness, response, and recovery stages and informed a proposed framework for future process review. Because no comparison group or standardized outcome measures were available, no conclusion can be drawn about the effectiveness or causal effects of the recorded response or recovery measures.
